# Modelling intercity accessibility surfaces through different transport modes in the Yangtze River Delta mega-region, China

**DOI:** 10.1016/j.dib.2018.07.054

**Published:** 2018-07-26

**Authors:** Lei Wang, Xuejun Duan

**Affiliations:** a Manchester Urban Institute, School of Environment, Education and Development, The University of Manchester, Manchester, UK; b Key Laboratory of Watershed Geographic Sciences, Nanjing Institute of Geography and Limnology, CAS, Nanjing, China

## Abstract

The paper presents the data that is related in the research paper entitled “High-speed rail network development and winner and loser cities in megaregions: The case study of Yangtze River Delta, China” (Wang and Duan, in press) [1]. This data article describes the modelling results of spatially continuous accessibility surfaces through transport modes of the highway, conventional rail and high-speed rail networks in the Yangtze River Delta mega-region, China. By using a door-to-door approach to integrate intra- and inter-city travel, the data is stimulated in the geographic information system environment. It is calculated by the datasets of transport networks, land-use types and transport speeds which are mainly collected from the OpenStreetMap and GlobeLand30 and relevant design specifications on transport infrastructures, respectively. The data is stored in raster format and provides high spatial resolution at 100 m. The data can be used as a baseline in the studies of transport economics and planning.

**Specifications Table**

**Table:** 

Subject area	*Human Geography*
More specific subject area	*Transport geography*
Type of data	*Raster (Geotiff)*
How data was acquired	*Using different public datasets such as OpenStreetMap and GlobeLand30 and relevant design specifications on transport speeds to simulate the surface of intercity travel time to Shanghai in the Yangtze River Delta mega-region.*
Data format	*Analysed*
Experimental factors	Dividing the study area into 34.81 million 100 m * 100 m grids; using ArcMap tools of cost distance analysis and network analysis; modelling door-to-door travel time to Shanghai in a geographic information system environment
Experimental features	*High-resolution data of accessibility surface for travelling by high-speed rail, conventional rail, and highways*
Data source location	*The Yangtze River Delta mega-region including Shanghai, Jiangsu, Zhejiang and Anhui provinces, China*
Data accessibility	*Data is available within this article in the link provided*
Related research article	*Wang L., Duan X.J., in press. High-speed rail network development and winner and loser cities in megaregions: The case study of Yangtze River Delta, China*[1]

**Value of the data**

• Demand for high-resolution, spatially continuous accessibility surface data is growing in transport planning.
• This data can be used for examining the spatial disparity of intercity accessibility at the regional scale.
• The data can be used as the baseline for transport upgrading by researchers in spatial econometric models to estimate the effects of high-speed rail development on the restructuring of economic activities.
• The accessibility change resulting from the highway, conventional rail to high-speed rail can be calculated and used to estimate the expanded influence area of the regional economic center.

## 1 Data

### 1.1 Transport network and land-use types

**Fig. 1:**
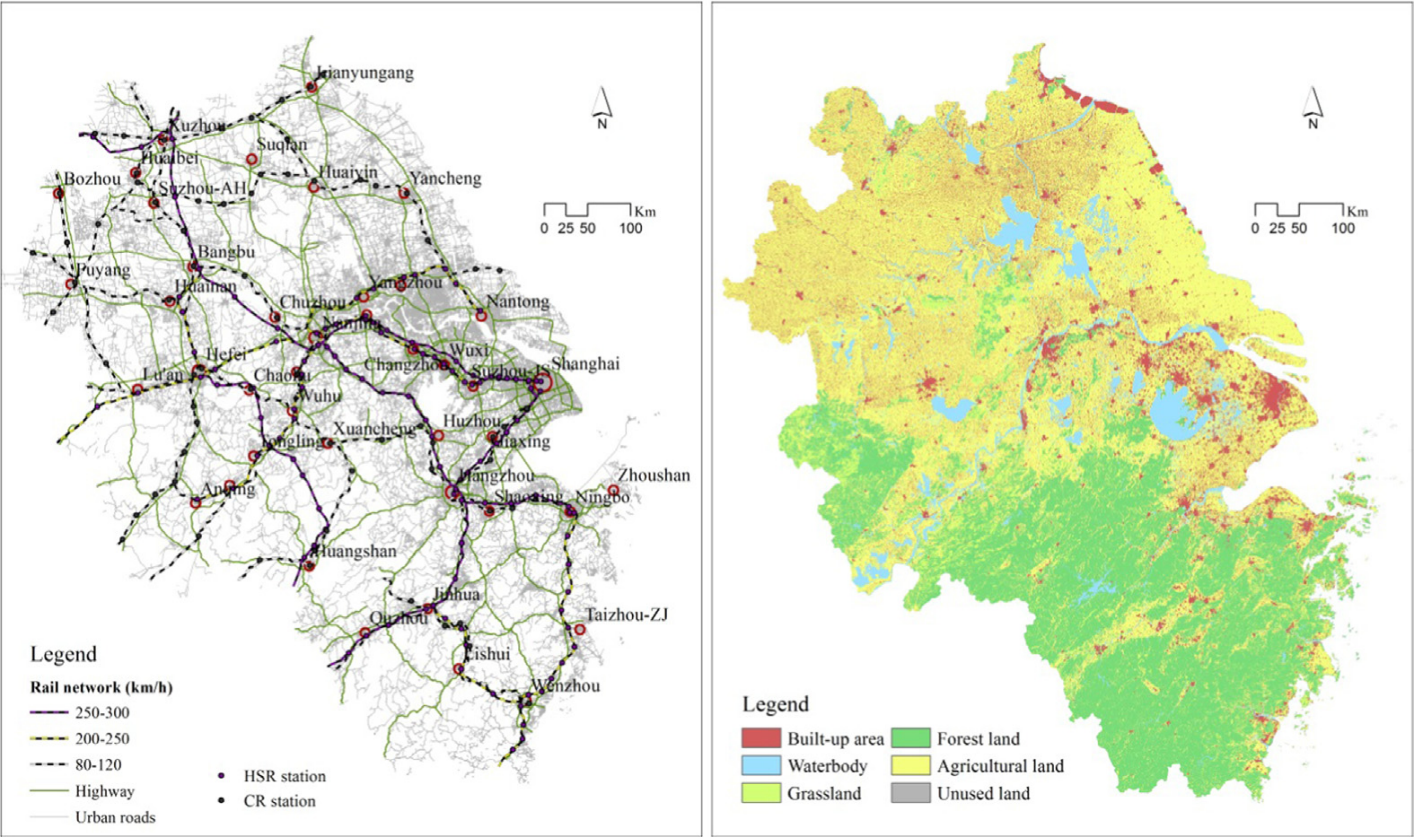
Transport and land use maps in the YRD.

The transport network data presented in this article is included in Fig. 1. The data mainly comes from OpenStreetMap and was further corrected with reference to the 2016 electronic atlas of Shanghai, Jiangsu, Zhejiang and Anhui in the Yangtze River Delta mega-region (YRD). The data includes types of the road like national and provincial highways, urban roads and streets, conventional rail (CR) and high-speed rail (HSR), railway stations and highway entrances. The lengths of HSR, CR and highway networks are 3788, 5235, and 13,607 km respectively. There total 192 stations among which 86 for HSR services only, 70 for CR services only and 36 for both services.

Land-use data in the YRD is extracted from the Global Land Cover Datasets (GlobeLand30). The original spatial resolution is 30 m, and land is classified with ten cover types. The data can be downloaded from the link: http://www.globallandcover.com/GLC30Download/index.aspx. According to the assessment of Brovelli, Molinari [2], the overall accuracy rate of the GlobeLand30 is higher than 80%. The data has been widely used in the studies of ecosystem services, climate change and regional planning in many countries [3], [4], [5]. In this data article, the land-use data is resampled into 100 m in line with our other datasets and reclassified into six types of built-up areas, agriculture land, grassland, forestland, waterbody and unused land (Fig. 1).

### 1.2 Inter-city accessibility of different transport modes

**Fig. 2:**
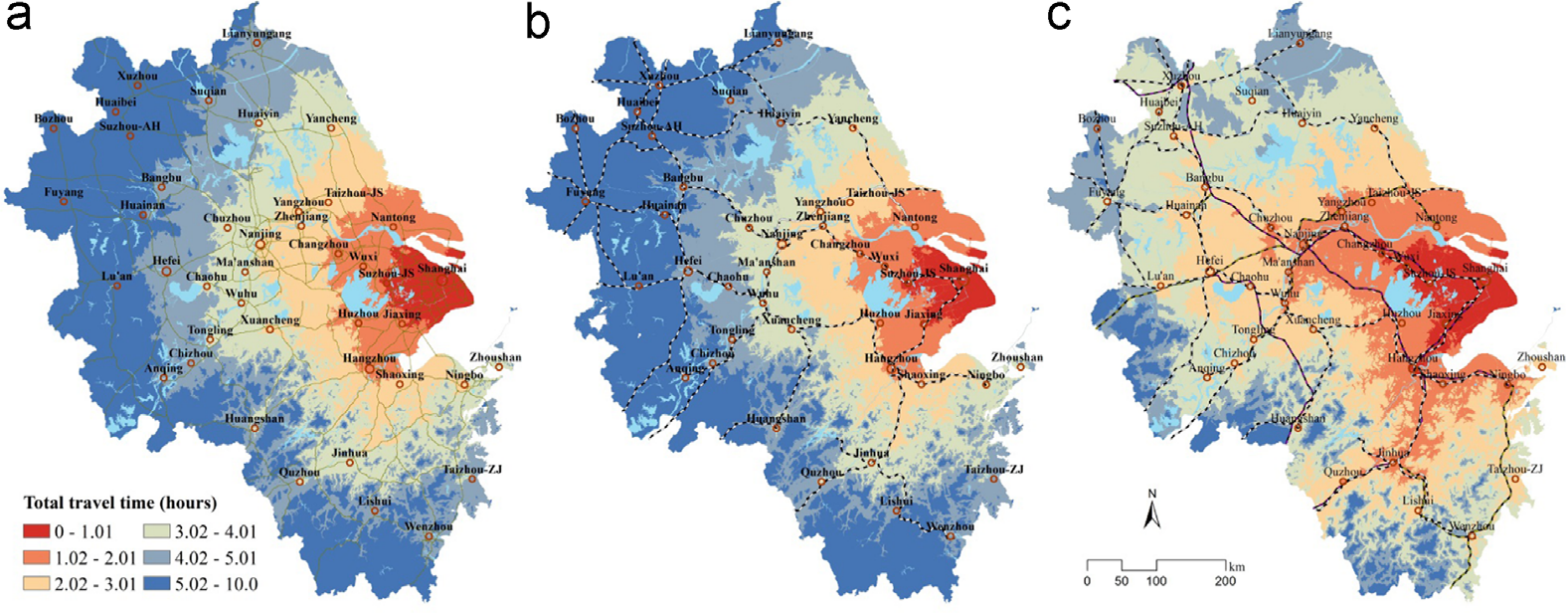
The travel time to Shanghai city centre mainly through highway (a), CR (b), and HSR (c) networks in the YRD.

The data presented for the intercity travel times to Shanghai through three transport modes of the highway, CR and HSR networks in the YRD (Fig. 2). The intercity accessibility is calculated through a door-to-door travel time measurement. The value of each grid is the travel time to Shanghai city centre. The data is included in three grid files. The spatial resolution of the data is 100 m. 41 sample locations of city centers at the prefectural and vise-provincial level illustrate that the modeling results exaggerate approximately 10% compared with the results of route query through Google Map (https://www.google.com/maps) and Baidu Map (https://map.baidu.com/).

**Fig. 3:**
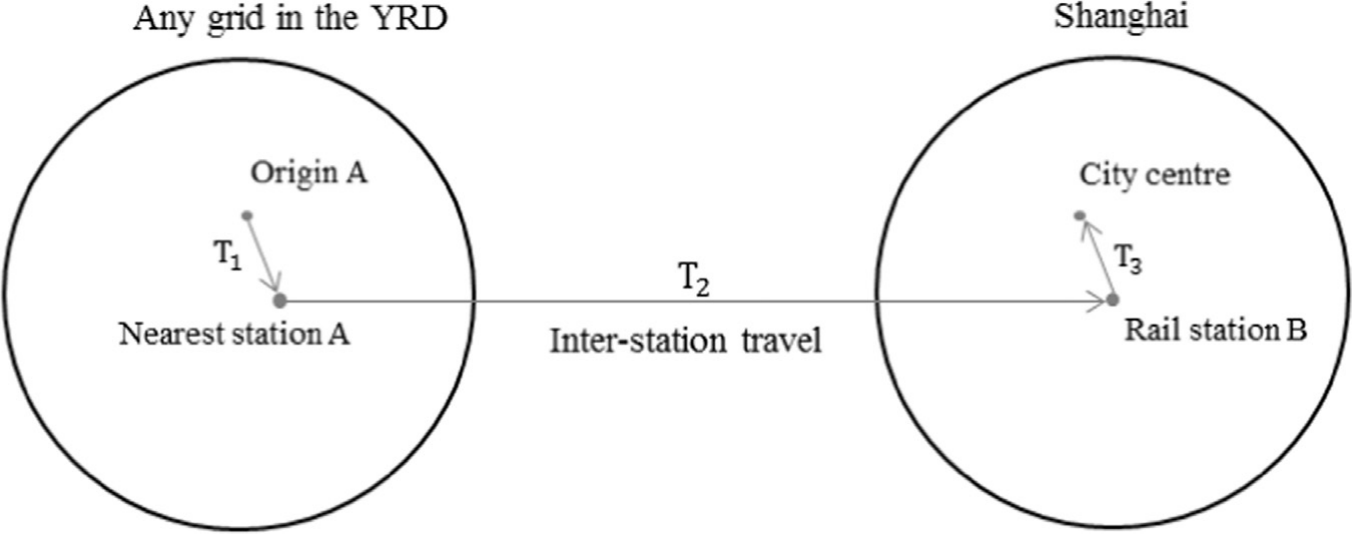
Conceptualising the inter-city travel time through a door-to-door approach.

## 2 Experimental design, materials, and methods

A door-to-door approach [6], [7], [8] is employed to calculate the minimal inter-city travel time to Shanghai in the YRD. If rail services are chosen, the travel time to Shanghai is calculated by three parts: (1) the intra-city travel time to the nearest station (T1); (2) the inter-station travel time to any of the three main stations including Shanghai, Shanghai South and Shanghai Hongqiao (T2); and (3) intra-city travel time after arrival from the station to Shanghai city centre (T3). The minimal total travel time is thus defined as:

(1) $$T_{A\to \mathrm{Shanghai}}={\mathrm{Min}\;(T}_{1}+T_{2}+T_{3})$$

The calculation of travel time through highway is quite similar to rail services. It needs to find the nearest highway enhance, travel to the next entrance, and then go to the city centre through the urban road network.

**Fig. 4:**
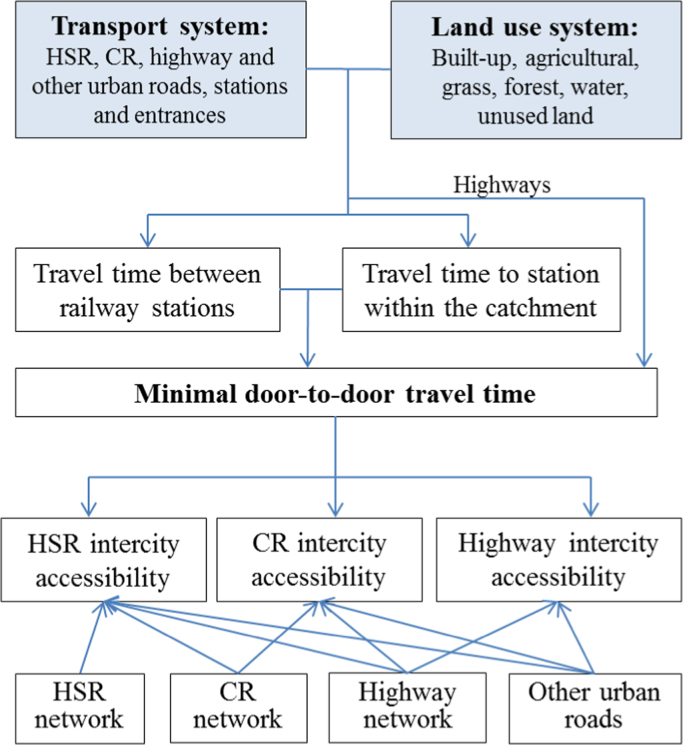
Data processing steps.

Fig. 4 outlines the steps of data processing to obtain inter-city accessibility by different transport modes. The intercity travel time is modelled in the Geographic Information System environment. A hybrid approach to integrating cost distance and network analysis is used to calculate inter-city travel time. More specifically, T_1_ and T_3_ are computed by cost distance analysis of the urban road network, whereas T_2_ is calculated by network analysis of the rail/highway network.

The travel speed of different transport modes and on different land-use types are outlined in Table 1. Relevant speeds are obtained through local design specification for train services, highways and other road networks and previous literature such as the study of Monzón et al. [9], Jiao et al. [10] and Wang et al. [6].

**Table 1:** Modelling travel speeds of different transport modes and land-use types.

Transport modes	Speeds (km/h)	Land-use types	Speeds (km/h)
HSR train I	300	Built-up area	20
HSR train II	250	Agricultural land	15
CR train	120	Grassland	10
Motorway	110	Forest land	2
National highway	80	Waterbody	5
Provincial highway	70	Unused land	5
City and county highway	40		
Streets	30		
Rail buffer of barrier	0.15		
Motorway buffer barrier	0.1		

Hence, the accessibility surfaces through the highway, CR, and HSR networks are obtained in the YRD (Fig. 3). Based on this dataset, the accessibility impact of HSR network development can also be stimulated by calculating the changes in travel time from the highway, and CR to HSR networks.
