# Supplementary material for: Modelling intercity accessibility surfaces through different transport modes in the Yangtze River Delta mega-region, China
Source: Data Brief. 2018 Jul 26;20:140–5. doi: 10.1016/j.dib.2018.07.054 (PMC6091314; doi:10.1016/j.dib.2018.07.054)
Supplement: Supplementary file 1 — Supplementary material [file mmc1.docx]

Conflict of interest

The authors declared no potential conflicts of interest regarding the research, authorship, and publication of this article.
